# Shrub encroachment into grasslands: end of an era?

**DOI:** 10.7717/peerj.5474

**Published:** 2018-09-05

**Authors:** Cho-ying Huang, Steven R. Archer, Mitchel P. McClaran, Stuart E. Marsh

**Affiliations:** 1 Department of Geography, National Taiwan University, Taipei, Taiwan; 2 Research Center for Future Earth, National Taiwan University, Taipei, Taiwan; 3 School of Natural Resources and the Environment, University of Arizona, Tucson, AZ, United States of America

**Keywords:** Landform, Crassulacean Acid Metabolism (CAM), Grazing, Long-term ground measurement, Remote sensing, Repeat photography

## Abstract

Shifts in the abundance of grasses and woody plants in drylands have occurred several times during the Holocene. However, our understanding of the rates and dynamics of this state-change in recent decades is limited to scattered studies conducted at disparate spatial and temporal scales; the potential misperceptions of shrub cover change could be remedied using cross spatiotemporal scale analyses that link field observations, repeat ground-level photography and remote sensing perspectives. The study was conducted across a semi-arid landscape in southern Arizona. Local data from long-term transects revealed three distinct chronological phases of shrub cover change: expansion (1961–1991, 0.7% y^−1^), decline (1992–1997, −2.3% y^−1^) and stabilization (1998–2012, 22–25% with no net cover change). Twenty-eight years (1984–2011) of broad-scale Landsat Thematic Mapper assessments confirm that shrub cover has been relatively stable in recent decades regardless of grazing regimes and landforms with the exception of the proliferation of succulents at lower elevations (verified by repeat photography acquired in 1987 and 2015) where the physical environment is the harshest, reflecting elevated temperature and winter precipitation deficit. Warmer, drier future climates are predicted to reduce woody plant carrying capacity and promote a shift to xerophytic succulents.

## Introduction

Ecological stability is generally defined as a system maintaining a dynamic equilibrium through time (Holling, 1973; Westman, 1978). When ecosystems are de-stabilized, directional changes in structure and function occur and new steady-state configurations may result (Folke et al., 2004; Scheffer & Carpenter, 2003). Grassland and savanna ecosystems characterizing many of the world’s drylands occupy about 40% of the land surface (Millennium Ecosystem Assessment, 2005; Reynolds et al., 2007) and play a crucial role in global biogeochemical, hydrological and energy cycles. They have been de-stabilized in recent history and transformed into deserts, shrublands or woodlands as palatable, mesophytic grasses and herbaceous vegetation are replaced by bare ground and unpalatable xerophytic shrubs (Sankaran, Ratnam & Hanan, 2004; Van Auken, 2000). This shift in plant life form and functional group composition alters primary production, nutrient cycling, land surface-atmosphere interactions and a myriad of related ecosystem processes (Eldridge et al., 2011) and services (Archer et al., 2017).

Although the proliferation of woody vegetation in drylands is recognized as having potentially important impacts on terrestrial carbon pools, the lack of information on the trends of shrub abundance is a major source of uncertainty in assessing how this vegetation change has influenced the carbon cycle (Asner et al., 2003; Browning et al., 2008). In North America, the destabilization of some grassland, savanna and shrub-steppe systems appears to have been initiated in the late 1800s and early 1900s; in other cases, the encroachment has been more recent; and in still other cases, woody plant abundance has changed little (see Barger et al. (2011) for a comprehensive summary). These contrasting realities have made it challenging to generalize about what factor or combination of factors might be driving (climate, land use, atmospheric chemistry) or constraining (geomorphology, soils) this vegetation change in a given locale or ecoregion (Archer et al., 2017). Projecting future consequences of woody cover (interchangeably used with “shrub cover” hereafter) change on ecosystem function will require knowledge of landscape features that make sites susceptible or resistant to shrub encroachment and where shrub cover in present-day landscapes lies relative to its maximum potential (Scholtz, Fuhlendorf & Archer, 2018). Presently, we have very little knowledge of the extent to which shrub communities developing on grasslands have reached their developmental potential, nor what that potential may be. This paper is a first step at addressing this knowledge gap.

Our understanding of how fundamental ecosystem processes change with herbaceous-to-woody state-changes is inconclusive (House et al., 2003); a synoptic perspective combining different observational approaches on changes in woody plant abundance across environmental gradients over large spatial extents and through time is necessary. One solution is to investigate time-series woody cover dynamics from permanent plots (Báez & Collins, 2008; Munson et al., 2012), a network of transects (Riginos et al., 2009) or repeat ground level photographs (McClaran, Browning & Huang, 2010). These provide detailed, high-resolution information, which can reveal trends in woody plant abundance. However, this type of data is seldom available and is highly limited in its spatial extent. Advances in remote sensing have the potential to overcome the spatial extent constraints inherent in field studies and encompass a wider range of conditions. Historical aerial photographs dating back to the 1930s and 40s make it possible to obtain broader coverage and quantify changes in the spatial pattern and extent of woody plant abundance (Laliberte et al., 2004; Fensham, Fairfax & Archer, 2005). However, poor or variable image quality, low resolution, and a lack of spectral information limit their utility in detecting woody plant canopies, and difficulties in automated processing limits the feasibility of their use for large areas. Utilization of contemporary satellite imagery to map the spatial variation of vegetation structural attributes (e.g., tree cover and height) over a vast region may be valuable for space-for-time substitution to investigate the relationships between vegetation state transitions and precipitation regimes (Hirota et al., 2011; Xu et al., 2016). However, the coarse data resolution of satellite imagery, limited ground-truthing, and lack of information on land use history make it difficult to identify causes for the transitions, which could vary from region to region (Synodinos et al., 2018). The legacy of decadal earth satellite observations, such as the Landsat Thematic Mapper (TM) Mission from 1984 to 2011, provide new opportunities to monitor dryland woody cover through time over vast areas. However, its broad spatial extent requires averaging across diverse landforms, soils and management units and its low resolution precludes quantification of shrub population structure, thus masking important constraints, drivers and trends. Integrating long-term field and satellite perspectives on woody cover change could provide the fullest amount of information about its trend through time. Coupling local, fine-scale ground data with broad-scale satellite imagery would offer an opportunity to provide robust regional perspectives on woody plant proliferation in drylands.

In this study, we used the 20,000 ha Santa Rita Experimental Range (SRER: https://www.ag.arizona.edu/srer/) in southeastern Arizona, USA as a model system to investigate the stability of woody plant cover in an era of climate and land use change. Shrub encroachment at this semi-desert grassland ecosystem began in the early 1900s and has been well-documented with repeat ground photography and transects (McClaran, Browning & Huang, 2010). Here, we integrate perspectives from long-term (1957–2012) field transect data and decadal repeat photography with 28 years (1984–2011) of satellite remote sensing to interpret shrub cover dynamics within the context of land use and topoedaphic factors. Our objectives were to (i) quantify rates, patterns and trends of shrub cover abundance in recent decades, and (ii) determine the extent to which biophysical setting and land use influenced on the spatiotemporal dynamics of shrub cover.

## Material and Methods

### Site description

**Figure 1:**
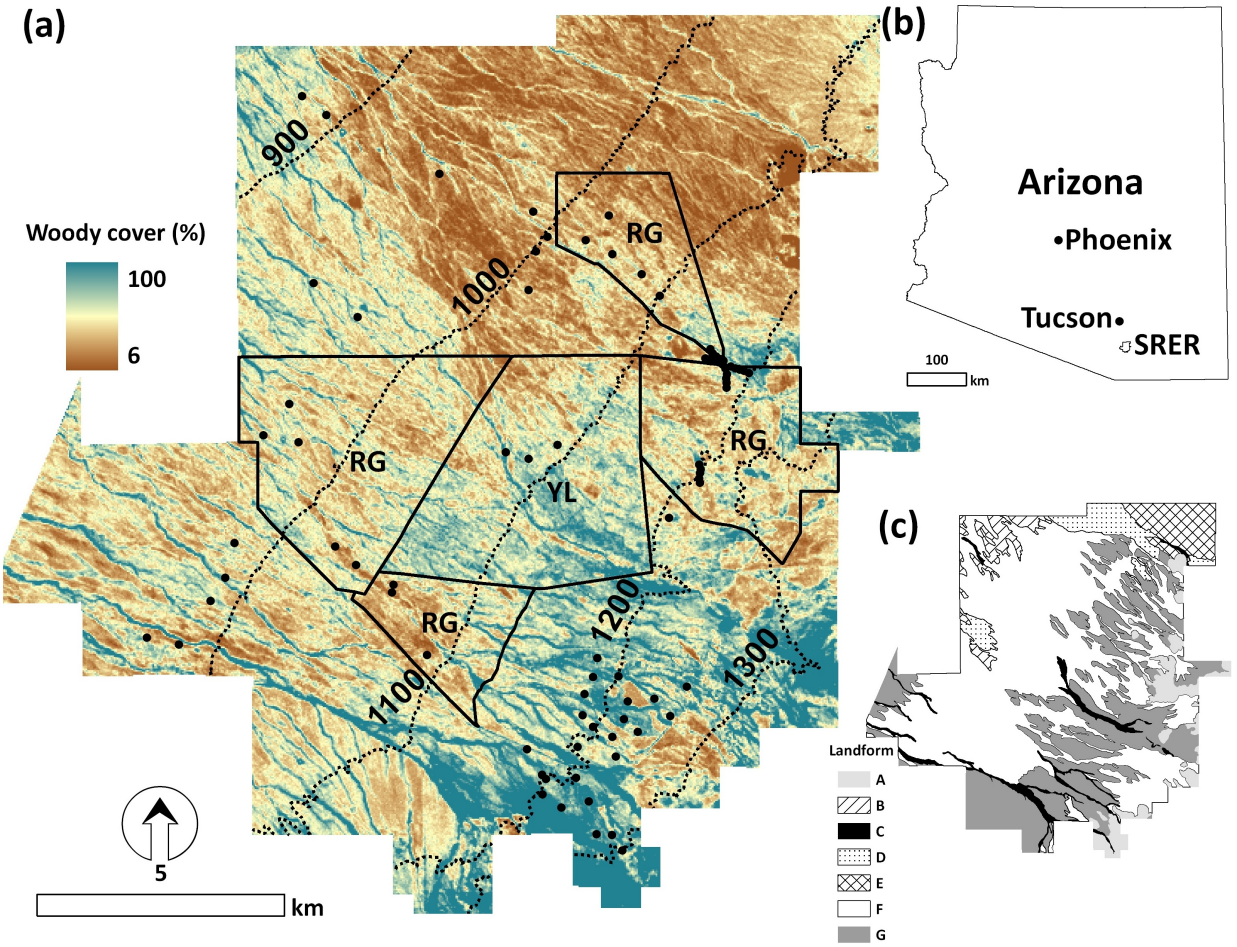
The study site for long-term shrub abundance monitoring. The 20,000 ha Santa Rita Experimental Range (SRER) showing (A) long-term (1984–2011) mean woody cover (derived from Landsat Thematic Mapper [TM] images) across an elevation gradient (meters, dotted irregular lines); (B) its location in Arizona; and (C) major landforms (see Table S1 for details). Black dots in (A) denote locations of long-term, repeatedly measured transects (*n* = 81). Irregular polygons indicate pastures with yearlong (YL) or rotational grazing (RG) livestock grazing regimes; areas outside these polygons received mixed grazing.

Our study site was located along the western edge of the semi-desert grassland region of the North American Sonoran Desert (31.83°N, 110.85°W) (Fig. 1). Established in 1902, the SRER is the oldest experimental range in the USA (Bagchi et al., 2017; McClaran, Browning & Huang, 2010). Annual precipitation of the Sonoran Desert is bimodal with a pronounced peak (>50% of mean annual precipitation (MAP)) in summer (July–September monsoon) and a lesser peak (∼30% of MAP) in late winter and spring (December–March) (Sheppard et al., 2002). Elevation (mean = 1,076 m a.s.l.; range 867–1,582 m a.s.l.) at this site is a rough proxy for climate, as MAP increases ∼37 mm per 100 m elevation gain, ranging from 305 mm to 489 mm. Mean annual temperature (MAT) is 17.9 °C at 1,310 m (Huang et al., 2007). A diversity of landforms occur within the SRER (Fig. 1C and Table S1). Physiognomy ranges from *Larrea tridentata* desert scrub at the lowest elevations to *Quercus* spp. woodlands at the highest. *Prosopis velutina* is the dominant shrub at 990–1,200 m elevation. Cacti (mainly *Opuntia* spp.) occur across the elevation gradient. The range of vegetation types within the SRER encompasses about 20% of the total amount in Southwest, and is representative of many arid and semi-arid regions of the Sonoran Desert (Lowry et al., 2007).

Prior to 1902 the southwestern USA experienced heavy, unregulated livestock grazing. Shortly after the SRER was established in 1902, livestock were excluded and then re-introduced in 1916. The site was then managed under yearlong (YL) livestock grazing until 1957 when various rotational grazing regimes were implemented. During 1972–2007, YL and the Santa Rita Grazing System (RG) have been consistently applied in one (1,855 ha, mean elevation (± standard deviation, sd) = 1,092 ± 35 m a.s.l.) and four (3,988 ha, 1,085 ± 106 m a.s.l.) designated pastures, respectively (Fig. 1A). Those receiving RG were grazed twice (March–October and November–February) during a three-year cycle with one-year rest between each utilization (Mashiri, McClaran & Fehmi, 2008). Remaining areas (hereafter “Mixed”) experienced various grazing regimes through time. Two wild fires occurred in 1989 and 1994 burning 144 and 1,368 ha, respectively (Huang et al., 2007). In addition, *P. velutina* within a 71 ha area were cleared in 1976 by chaining. The main focus of this study was to investigate the recent trend of woody cover abundance afterseveral decades of woody plant encroachment. Therefore, these burned or shrub cleared areas of known destructive changes were excluded from our analyses since the systems may be still in the recovery phase.

### Long-term field and climate records

Projected shrub cover (defined as the planometric view of percentage of ground surface covered by shrub canopy) between 1957 and 2012 was assessed via archived data from permanent line transects (30.5 m length; *n* = 132) representing a range of topo-edaphic settings. We used 81 transects widely distributed across the SRER, after excluding transects experiencing disturbance and those with prolonged data gaps (Fig. 1A). Directional changes in cover were visually identified and trend analysis was applied to verify their statistical significance and to quantify the rate of projected woody cover change. Other metrics derived from the transects included frequency of occurrence (percentage of transects with a given species) and relative percent woody cover (relative to a base year).

Long-term (1895–2011) precipitation and temperature data for summer (April–September) and winter (from previous October to current March) were acquired from the Parameter-elevation Regressions on Independent Slopes Model (PRISM, McClaran & Wei, 2014; Stoklosa et al., 2015). Summer and winter precipitation and temperature time-series records were based on years coinciding with those for which field transect data on shrub cover was available (1957 to 2012). Longer-term climate trends were assessed by computing annual deviations from mean precipitation and temperature (1895–2012) for the summer and winter seasons.

### Remote sensing

Greenness in drylands derived from pre-monsoon Landsat imagery can be treated as live woody cover since herbaceous plants are mostly senescent and only deep-rooted shrubs can sustain green leaves through the support of relatively stable ground water (Asner et al., 2003; Scott, Cable & Hultine, 2008). The semi-arid environments of southern Arizona generally exhibit the lowest levels of herbaceous cover in May-June; and green vegetation signals derived from satellite data during this period are almost exclusively from shrubs and cacti (Huang et al., 2007). We therefore utilized 28 sets of the pre-monsoon Landsat TM images (Table 1, obtained from http://glovis.usgs.gov/) with minimum cloud contamination to quantify temporal changes in woody cover from 1984–2011. These images contain six 30 m visible, near infrared and shortwave infrared bands, and one thermal band. All images were geo-registered (the Universal Transverse Mercator zone 12 N, World Geodetic System 1984) by the provider prior to acquisition and converted to surface reflectance using ACORN (Atmospheric CORrection Now v. 6, ImSpec LLC, Palmdale, CA, USA).

**Table 1:** Satellite imagery selected for dryland shrub monitoring. Image dates are of the Landsat Thematic Mapper utilized in the study.

Year	Date		Year	Date	
	May	June		May	June
1984	26		1998		02
1985		14	1999		21
1986	16		2000		07
1987	03		2001		10
1988	21		2002		13
1989		09	2003	15	
1990	27		2004		02
1991	14		2005		05
1992	16		2006	23	
1993		04	2007	26	
1994		07	2008	28	
1995		10	2009	31	
1996	27		2010		03
1997		15	2011	21	

A probabilistic spectral mixture analysis model was used to unmix fractional green woody vegetation cover from each pixel on all images, and rectified to ground-measured projected woody cover by referring to Huang et al. (2007). The model is ideal for use in complex environments where sub-pixel cover variation is high. We applied trend analysis on pre-monsoon time-series Landsat imagery in this study to delineate shrub cover dynamics for each individual pixel and the overall trend on the SRER. However, our field observation indicated that episodic pre-summer monsoon rainfall may initiate the growth of green vegetation (especially for herbaceous plants) and falsely amplify estimates of woody cover locally, making the year-to-year comparisons inconsistent. Therefore, for the pixel-based analysis, a correction procedure Eq. (1) was carried out to reduce the uncertainty. The procedure was not used to derive the actual trend but an apparent directional change:

(1) \begin{eqnarray*}\text{Adjusted trend}=\text{trend}\times \text{model fitness}\;({R}^{2}).\end{eqnarray*}

Each pixel was classified into one of three woody encroachment categories (relative to the preceding date): increase, stable or decrease. In order to set up reasonable thresholds to define overall trends in these categories, we first estimated the baseline condition of mean annual woody cover increase based on the visually identifiable period of regional woody plant encroachment. To make this value comparable to the adjusted woody cover trend, we then transformed the mean annual field woody cover increase by multiplying with mean model fitness (*R*^2^, Eq. (1)). This scaling procedure reduced the actual trend value but did not influence the interpretation since the remotely sensed trends were also modified by their own spatially corresponding *R*^2^. Assumptions were also made that the same degree of decrease (less than the negative adjusted trend) were recognized as woody cover “decline” sites; areas with trends in between “increase” and “decline” were labelled as “stable”. Regions with apparent changes (increase or decline) in woody cover were further investigated by examining spatiotemporally corresponding field repeat ground-level photography from the SRER Digital Archive (https://www.ag.arizona.edu/srer/) and carrying out site visits.

### Woody cover trend analysis

Change or stability in woody cover was assessed in relation to grazing regimes (YL, RG, Mixed) and landforms. Definitions of “stability” are varied and depend on the structural attributes or functional processes of interest and spatial/temporal scales (Grimm & Wissel, 1997). Here, we define stability in the context of grassland-to-shrubland state-change as the persistence of a set amount of woody cover for a sustained period of time (the “stable” pixels). Desert shrublands are generally situated on flat or gently sloping terrain (Brown, 1994). Since the majority of slope inclinations on the SRER were ≤3° (77% of the land area), slope aspect and inclination were not assessed. Spatial layers of the grazing regime (Fig. 1A) and geomorphic landforms (Fig. 1C) were obtained from the SRER Digital Archive.

## Results

### Field woody cover and climate trends

**Figure 2:**
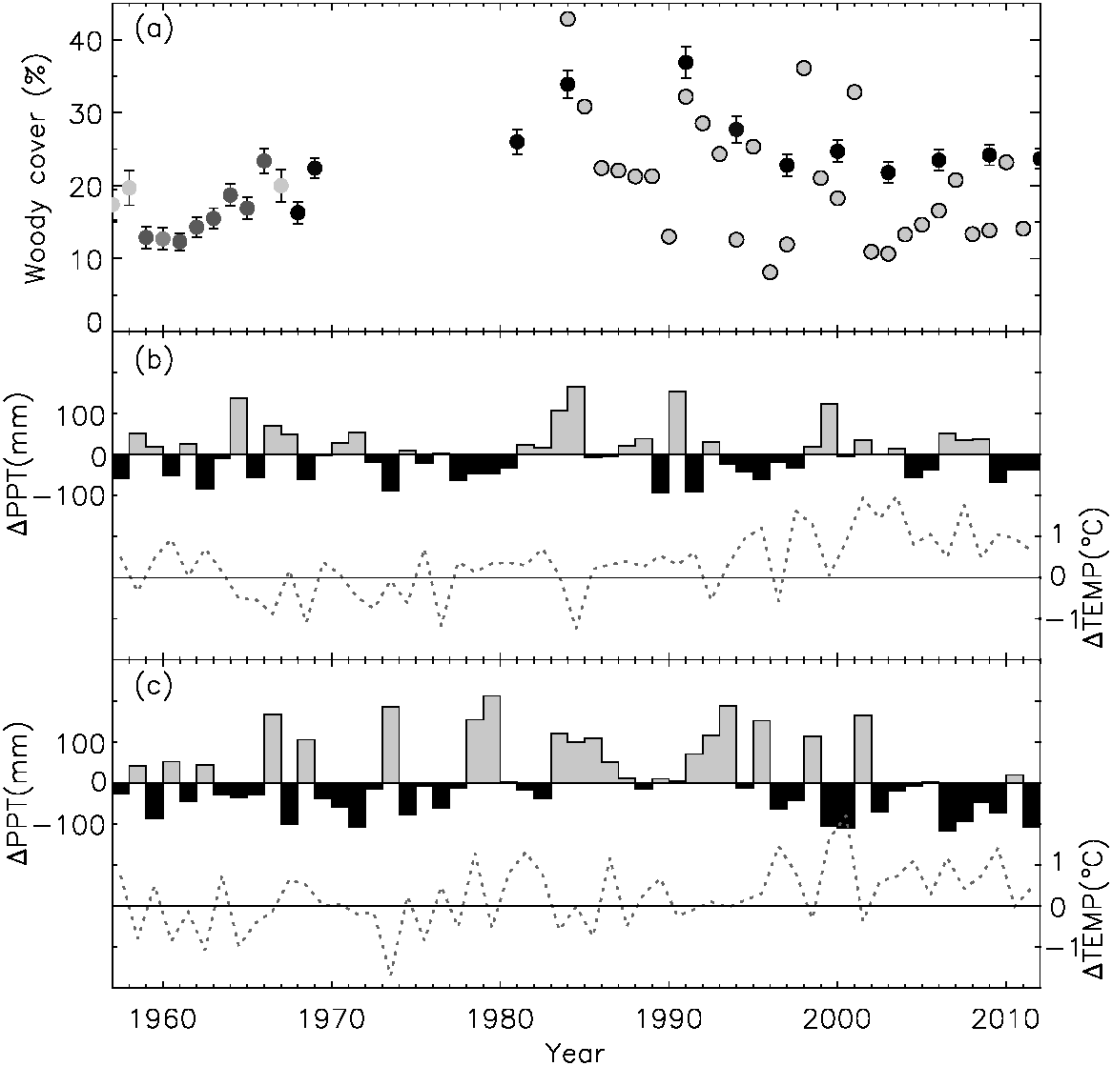
Temporal dynamics of woody cover and climate. (A) Trends of mean (+ standard error) woody cover at the SRER measured on field transects (gray monochromatic circles) and from satellite images (light gray outlined circles). Darkness of field circles corresponds to the sample sizes that were included in the calculation of the mean, varying from 37 (light gray) to 81 (black) transects. Detailed statistics are presented in Table S2. Deviations of from long-term (1895–2011) mean (B) summer (April–September) and (C) winter (October–March) precipitation (ΔPPT [bars]; 236 mm and 155 mm, respectively) and temperature (ΔTEMP [lines]; 23.8 °C and 12.7 °C, respectively) are also shown.

A comparison of 1961 (the lowest starting point) and 2012 (end point) field transect data indicated little change in shrub cover over the 51 y period (0.2% y^−1^ (*p* = 0.002) or total 11.5% with the mean (± sd) woody cover of 22.4 ± 6.1%, Fig. 2A). However, this coarse temporal assessment masked three visually distinct phases: (i) an encroachment phase (1961–1991 (peak shrub cover = 36.9%), 0.7% y^−1^ (*p* < 0.0001, *n* = 12), 24.6% increase with the mean (± sd) woody cover of 21.4 ± 7.3%), followed by (ii) an abrupt decline phase (1992–1997, −2.3% y^−1^ (*p* = 0.1, *n* = 3) or 14.1% loss of shrub cover with the mean (± sd) woody cover of 25.2 ± 2.4%) and (iii) a stabilization phase (1998–2012, 0.0% y^−1^ (*p* = 0.63, *n* = 6), shrub cover fluctuated between 22% and 25% with no net change; mean (±  sd) woody cover = 23.6 ± 1.0%).

Centurial (1895–2012) mean summer and winter precipitation averaged 236 mm and 155 mm, respectively, with temperature averaging 23.8 °C and 12.7 °C, respectively. Deviations from these long-term means during the 1961–2012 period of field transect data are shown in Figs. 2B and 2C. Summer and winter precipitation fluctuated during this early to middle portions of this period, but there has been a trend toward less winter precipitation since about 1999 when winter precipitation has declined an average 34.7 mm (22.4% decline from the long-term average). Temperature also fluctuated around the long-term mean until about 1994 at which point it has been consistently higher, particularly in summer.

### Remote sensing of woody cover trends

**Figure 3:**
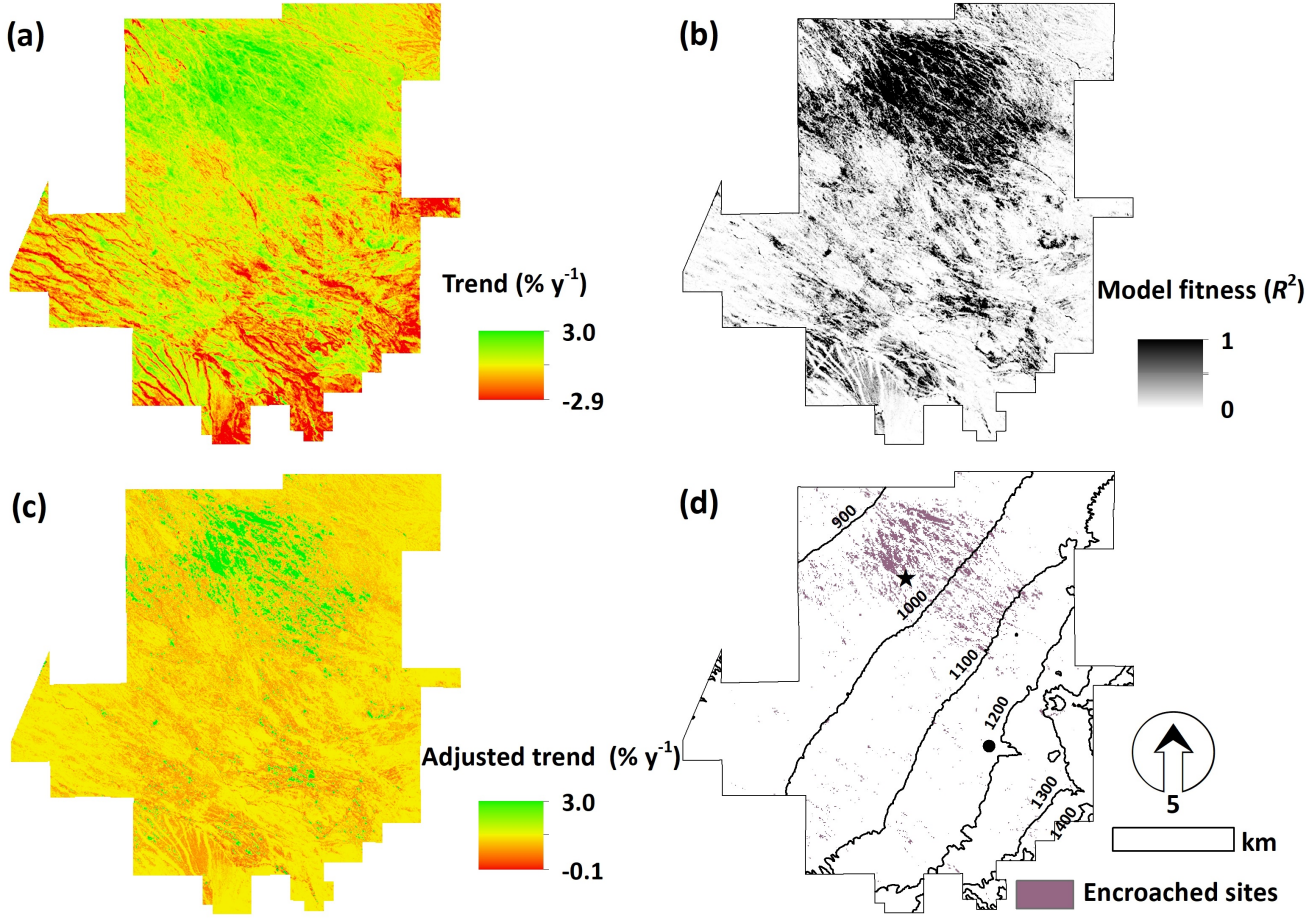
Remotely sensed trend analysis of dryland woody cover. (A) Spatial pattern of rates of change in woody cover (% y^−1^) on the SRER over a 28-year period (1984–2011) derived from time-series dry season Landsat images and (B) corresponding model fitness (*R*^2^). (C) Adjusted rates of woody cover change after suppressing signals from noisy pixels with a correction procedure. (D) Sites where rates of woody plant encroached exceeded 0.1% y^−1^. The star and circle in (D) denote the locations of ground-level repeat photo stations shown in Figs. 4A and 4B, respectively. The unit of the contour lines is in meter.

Mean (± sd) woody cover across SRER was 20.6 ± 7.9% over the 28-year satellite record (Fig. 1A), which was slightly lower (one-way analysis of variance, *p* = 0.06) than the 26.6 ± 5.3% estimates from field transects during the corresponding time period (1984–2011). Woody cover was generally highest along arroyos and at upper elevations. Although mean woody cover values fluctuated from year to year, a tendency toward recession (about 14% decline and 0.5% y^−1^ (*p* = 0.01)) with lower year-to-year variation since 1984 was observed (Fig. 2A). The areas exhibiting increases in woody cover during this time-period were mainly (91.7%) located at lower elevations (e.g., 867–1,100 m, Fig. 3A). Elevations above 1,300 m a.s.l. characterized by relatively high woody cover (Fig. 1A) generally experienced net decreases between 1984 and 2011. However, the apparent trend of woody cover net decreases at upper elevations was significantly reduced (Fig. 3C) after the correction (Eq. (1)) for model fitness (Fig. 3B) to minimize the noise induced by pre-monsoon intermittent rainfall triggered vegetation green-up.

**Figure 4:**
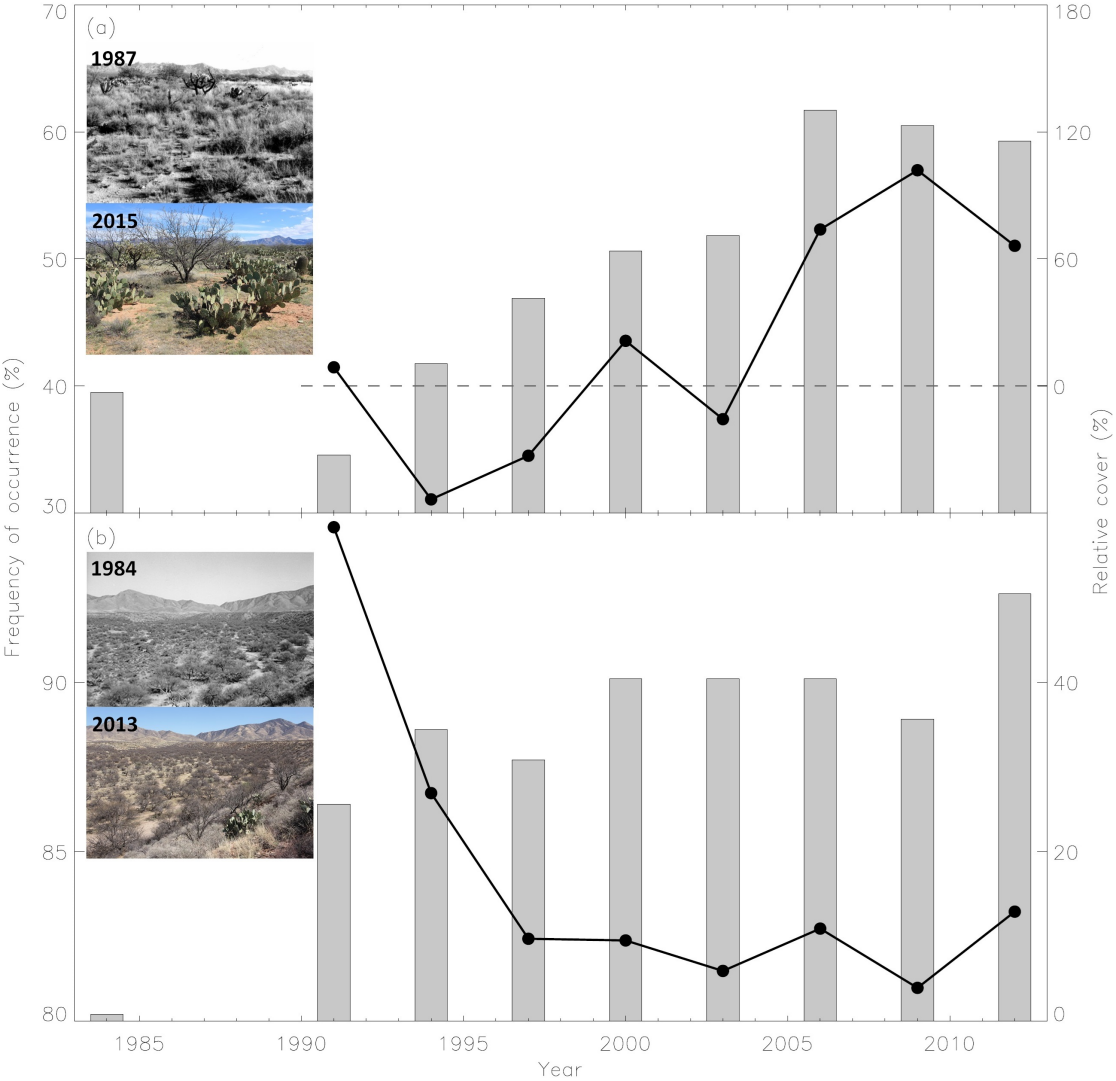
Abundances of *O. engelmannii* and *P. velutina* through time. Frequency of occurrence (% of total transects with the observation of certain species) of (A) the dominant CAM succulent *O. engelmannii* and (B) dendritic C_3_ shrub *P. velutina* along field transects (bars, *n* = 81; left-hand *y*-axis), and their abundance (% cover, solid line, right-hand y-axis) relative (the dashed line) to those in 1984 (mean cover of *O. engelmannii* = 1.2%; *Prosopis velutina* = 14.0%). Inset photos are SRER repeat photography station #301 (31.88°N, 110.88°W) and #222 (31.82°N, 110.84°W) (denoted by the star and circle, respectively, in Fig. 3D; see https://www.ag.arizona.edu/srer/photos.html for detailed site description) acquired in late 1980s and 2000s illustrating the proliferation of cacti and stabilization of shrubs, respectively, during the Landsat TM observation period. Photo credits: Robert Buttery (1980s) and Mitchel McClaran (2000s).

A threshold of ±0.1% was applied on the adjusted trend. The value was set by multiplying the trend line of 0.7% y^−1^ during the field woody cover encroached period (1961–1991) (Fig. 2A) by the mean model fitness (mean *R*^2^ = 0.14) (Fig. 3B) of remotely-sensed trend estimates. We note that the adjusted trend of decline was negligible since the total maximum shrub cover decline was <3% since 1984 and no spatially ostensible clusters were observed. Woody cover on the majority (96%) of SRER land surfaces was stabilize (mean adjusted woody cover trend ± sd = − 0.01 ± 0.02% y^−1^) (Fig. 3C); sites exhibiting woody proliferation were spatially clustered at elevations below 1,100 m a.s.l. Examination of spatiotemporally corresponding data from field repeat ground-level photography stations and species occurrence frequency transects (Figs. 3D and 4A) revealed a strong trend of cacti (mainly *Opuntia engelmannii*) proliferation in terms of its frequency of occurrence and % cover relative to that in 1984. Over the same period, these metrics of shrub (mainly *P. velutina*) abundance declined and then stabilized (Fig. 4B).

### Grazing regimes and landforms

Directional trends in woody cover abundance were minimal to non-existent on all landforms (mean adjusted woody cover trend = −0.010–0.003% y^−1^ (or −0.27–0.08% of shrub cover during the satellite observation period), sd = 0.008–0.069% y^−1^) and grazing regimes (mean adjusted woody cover trend = −0.011–0.002% y^−1^ (−0.30–0.05% total), sd = 0.039–0.065% y^−1^). An evaluation of grazing regimes and landforms controlling for elevation also failed to reveal pronounced woody cover trends (mean adjusted woody cover trend = −0.014–0.005% y^−1^ (−0.38–0.14% total), sd = 0.008–0.072% y^−1^). Differences among these groups were negligible with no biological significance; therefore, no statistical analysis was performed.

## Discussion and Conclusions

Over the past century, one of the most significant land cover changes in drylands has been the proliferation of trees and shrubs at the expense of perennial grasses. This shift in plant life composition has been widely reported qualitatively (Van Auken, 2000) and quantitatively (Asner et al., 2003; Laliberte et al., 2004). However, the scarcity of historical data in many places of the world often restricts the assessment of vegetation dynamics to only two points in time with no accounting of changes that may have occurred between those points. Long-term continuous cross-scale observations with standardized protocols are seldom available which makes it extremely difficult to quantify rates and patterns of shrub cover change and ascribe causal explanations. These challenges highlight the uniqueness of SRER and make it one of the most suitable model sites for studying woody plant encroachment in the Sonoran Desert. Based upon the long-term field observations encompassing many points-in-time, we find that change in mean shrub cover was not uni-directional but consisted of expansion, decline and stabilization phases. Wall-to-wall, broad-scale satellite assessments verified the stasis of woody plant encroachment in recent decades with the exception of the proliferation of succulents confirmed by long-term, on-site repeat ground-level photography at lower elevations where the physical environment is the harshest with the highest air temperature and lowest winter precipitation.

### Field observed woody cover stability

A large body of literature exists indicating that woody plant encroachment in drylands to replace grasslands by shrublands seems to be a norm in the arid and semi-arid environments (Archer et al., 2017). However, continental-scale analyses indicate that maximum woody cover is constrained by MAP (Sankaran et al., 2005; Scholtz, Fuhlendorf & Archer, 2018). Our data indicate that woody cover at the SRER, which had commenced in the early 1900s, hit that upper limit by the time satellite imagery was available in the 1980s, is now fluctuating in response to interannual and seasonal variability in precipitation, and is showing signs of responding to recent declines in annual precipitation and increases in temperature. In addition, the resilience of some transition-prone desert grasslands could reverse the prevailing trend of woody plant encroachment with the relaxation grazing (Bestelmeyer et al., 2013).

Our network of repeat ground measures revealed that the dynamics of woody cover abundance has not been uni-directional over the past 56 years. Three distinct trends of woody plant abundance were delineated within the observation period. In the first 35 years (1957–1991) with no pronounced evidence of climatic anomalies (Figs. 2B and 2C), mean woody cover increased steadily (Fig. 2A), which agreed with patterns observed from other bioclimatically similar sites (Peters et al., 2015). Potential mechanisms that triggered the encroachment of woody plants may be related to a combination of changes in biotic (inter- and intra-specific competition) and abiotic factors (land use, climate, increased atmospheric CO_2_ concentration, topo-edaphic characteristics and nutrient flows), interacting with changes in disturbance (fire, grazing/browsing) regimes (Klausmeier, 1999; Archer et al., 2017). Comparing the long-term field transect and meteorological data, we found that the rapid increase of shrub cover from 1981 (26%) to 1991 (36.9%) occurred in conjunction with above-average precipitation in both summer and winter during the 1980s (Fig. 2). The end point ostensibly indicates the maximum climatic carrying capacity of woody plant abundance of this bioclimatic region (Sankaran et al., 2005). The second stage (1992–1997) showed abrupt decline in woody cover, which was in-sync with the trends of two dominant species (*Isocoma tenuisecta* and *P. velutina*, McClaran, Browning & Huang, 2010). Mean woody cover had dropped to the level (∼20%) close to 1960s. Drivers resulting in the sudden reduction of woody cover abundance during this time-period remain unknown. This may reflect natural turnover for relatively short-lived, suffruticose woody species such as *I. tenuisecta* (McClaran, Browning & Huang, 2010); for long-lived arborescent species such as *P. velutina*, it may reflect the onset of density-dependent regulation (Browning et al., 2008). Woody plants may have ‘overshot’ the climatic carrying capacity by the 1990s, with subsequent mild summer droughts and the onset of elevated summer and winter temperatures (Figs. 2B and 2C) bringing cover levels back in line.

After the rapid period of woody cover decline, consecutive years (1998–2012) of drought occurred, especially during the winter months. Above-average temperature also occurred during this period. These regional meteorological records were in-sync with the broader trend in “global-change-type droughts” which have caused profound impacts on vegetation (Allen, Breshears & McDowell, 2015). Dryland plants live on the brink of their physiological limits for water and temperature stress; alteration of the climate region would impact the abundance of species substantially (Munson et al., 2011; Archer & Predick, 2008, but see Ulrich et al., 2014). Elevated woody plant mortality rates in response to drought have been doumented in the Southwest in the recent decade including Colorado (Miriti et al., 2007), Mojave (Hamerlynck & McAuliffe, 2008) and Sonoran (Bowers, 2005) Desert shrublands. These droughts had little impact on the persistence of shrub cover at the SRER, however. Based on the Sankaran et al. (2005) model for sites in Africa, MAP during this period could potentially sustain ∼34% of woody cover, which is 10.4% more of the mean value measured at SRER. Since no intense perturbations such as wildfire, heavy grazing or shrub management occurred during this period, the stasis of shrub cover below this upper limit may reflect constraints imposed by temperature and winter precipitation deficits. It has been proposed that plant diversity and ecosystem multifunctionality peak at intermediate levels of woody cover in global drylands (Soliveres et al., 2014). However, it is not clear where, along the encroachment-stabilization continuum observed on SRER this peak might be. The highly stable relative woody cover of the study region in the recent decades (90.8–97.4%, analysis not shown) could have negative impacts on plant diversity and multiple ecosystem processes in a semi-arid setting (Soliveres et al., 2014). Knowledge of controls over shrub demography (e.g., patterns of shrub recruitment, growth and mortality), along with knowledge of shrub longevity, will be needed to predict future trends in shrub cover under changing environmental conditions.

### Remote sensing of woody cover trend

One advantage of remote sensing is to provide broad-scale and spatially continuous tracking of woody cover through time. In the Sonoran Desert, the orographic effect plays a crucial role in ecosystem productivity where more precipitation is received at higher elevation (Whittaker & Niering, 1975). The tendency was clearly observed at SRER (Fig. 1A). The notable exception were linear aggregations of shrubs along intermittent drainages (arroyos) receiving precipitation supplements in the form of water running off neighboring and upslope locations. These precipitation supplements make arroyo settings more mesic than other landforms and capable of supporting more shrub cover and biomass (Sponseller & Fisher, 2006; Miriti et al., 2007). Satellite-sensed estimates of woody cover through time generally agreed with, but were relatively lower (with weak statistical significance) than those from ground transects (Fig. 2A). This could reflect differences in sampling spatial coverages, and the low level of vegetation in the northern part of SRER was disproportionately sampled less (Fig. 1A). Although the two woody cover transition classes recognized on transect data (decline and stabilization) were not apparent in the coarser-resolution satellite time-series (Fig. 2A), the overall trend of a slight decline is consistent with field observations. This overall decline may be associated with consecutive years of winter precipitation shortage (Moreno-de las Heras et al., 2015) in concert with elevated temperature (Figs. 2B and 2C).

This study highlights the strength of remote sensing techniques to produce spatially extensive information across different land uses, which is not feasible for a field inventory program even at this well-managed experimental site. A correction procedure Eq. (1) was used to suppress the noise (rapid green vegetation onset) that was induced by the small amount of pre-monsoon rainfall (Figs. 3B and 3C). It was necessary to apply this noise reduction method to be able to consistently observe the sites through time and avoid the potentially confounding effects of pre-monsoon precipitation (Fig. 3D). The trade-off, however, is that subtler trends (Fig. 2A) may be masked. By examining the adjusted woody cover with the suppression of time-series data variation, we found that no noticeable trend in shrub cover for the majority of the 20,000 ha study area regardless of grazing practices and/or landforms with/without the control of elevation, which is a proxy for climate. The lack of trend and lack of grazing and landform influences supports the notion that shrub encroachment on the site had reached its apex and ‘carrying capacity’ by the time of the 1980s satellite imagery. This interpretation from broad scale satellite data is consistent with local high-resolution ground transect data and repeat aerial photography (Browning et al., 2008). With the advances of recent cloud computing (Hansen et al., 2013) and availability of field repeat photography for validation, we might be able to achieve a global-scale high spatial resolution mapping and monitoring of woody cover change in drylands.

### Succulent plant proliferation, the new trend?

Spatiotemporal dynamics of dryland vegetation will be influenced by changes in climate and fire regimes, and small animal populations, intensification of land use and/or increase in atmospheric carbon dioxide (Archer et al., 2017). However, consequences are extremely challenging to be generalized mainly due to the complex interactions among historical legacies (e.g., climate and past perturbations) and contemporary conditions; and resource re-distribution by fluvial, aeolian and/or animal vectors affected by topoedaphic attributes (Peters et al., 2006). Despite these potential local differences, our 28-year synoptic observations combining ground and satellite measurements revealed that woody cover in recent decades has stabilization at the study site. The most notable change in the recent decades has been a cover increase at lower elevations (Figs. 3C and 3D). With the confirmation from two independent field observations and site visits in the spring of 2012 (M McClaran pers. obs., 2012/4/24), we determined that these areas were associated with the proliferation of cacti (*Opuntia* spp.). The proliferation of cacti might reflect elevated temperature and winter precipitation deficits (crucial for deep-rooted woody plant growth (Snyder & Tartowski, 2006)) in recent decades (Fig. 2). These changes may further reduce the carrying capacity for dendritic C_3_ shrubs such as *P. velutina* (Fig. 4B) and promote a shift to xerophytic CAM succulents (Fig. 4A). A similar trend has also been observed within the Sonoran Desert region (Munson et al., 2012). Paleoecological records from packrat middens and fossils indicate massive die-off of long dominant *Juniperus osteosperma* and the replacement of *Pinus edulis* after prolonged, severe drought during 1250–1288 in northeastern Utah (Gray et al., 2006). This radical level of system alteration has not yet occurred at our site. However, the climate has become warmer and drier in Southwest since the mid-1990s (Overpeck & Udall, 2010). If this trend continues, dendritic shrubs that have displaced mesophytic grasses may themselves be replaced by xerophytic succulents. Such a change would have profound implications for ecosystem carbon pools and fluxes, phenology, biodiversity and land surface-atmosphere interactions.

##  Supplemental Information

10.7717/peerj.5474/supp-1 Supplemental Information 1 Supplementary Information Major landforms of the study site. The landform codes correspond to Fig. 1. Terms with numerical superscripts in the Name column are defined in the Description column.

## Additional Information and Declarations

### Data Availability

The following information was supplied regarding data availability: Field repeat ground-level photography: https://www.ag.arizona.edu/srer/photos.html.
