# Supplementary material for: Shrub encroachment into grasslands: end of an era?
Source: PeerJ. 2018 Sep 5;6:e5474. doi: 10.7717/peerj.5474 (PMC6129137; doi:10.7717/peerj.5474)
Supplement: Supplemental Information 1 — Major landforms of the study site. The landform codes correspond to Fig. 1. Terms with numerical superscripts in the Name column are defined in the Description column. [file peerj-06-5474-s001.docx]

**Supplementary Information**

**Table S1.** Major landforms of the study site. The landform codes correspond to Fig. 1. Terms with numerical superscripts in the Name column are defined in the Description column.

| Code | Name |  | Description |
| --- | --- | --- | --- |
| A | Hills^1^ and mountains^2^ (igneous and sedimentary rock) |  | ^1^Surface rising as much as 300 m above the surrounding.  ^2^Rising > 300 m. |
| B | Basin floor (alluvium, middle [25K-300K/400K] to early [300K/400K - 1M/2M] Pleistocene age)^*^ |  | Nearly level, lower-most part of intermontane basins including all the alluvial, eolian, and erosional landforms below the piedmont slope. |
| C | Inset fans^3^, stream terraces^4^, flood plains/drainages^5^ (alluvium of Holocene [10K-12K] age) |  | ^3^An ephemeral stream flood plain rather broad in area incised in alluvial fans or fan terraces; a barren channel with extensive breadth covering a minor portion of its surface.  ^4^One of a series of levels in a stream valley mostly paralleling the stream without flooding.  ^5^The nearly level plain bordering a stream and subject to inundation under flood-stage conditions. |
| D | Fan terraces^6^ or alluvial fans^7^ (alluvium from limestone sedimentary rock of Holocene or late Pleistocene [10K/12K - 25K] age) |  | ^6^General term for landforms that are remaining parts of older fan landforms, such as alluvial fan.  ^7^Low, outspreading mass of loose soil and rock material, commonly with gentle slopes that are shaped like an open fan or a segment of a cone, deposited by water at the place where it issues from mountains. |
| E | Fan terraces or alluvial fans (alluvium from limestone sedimentary rock of middle Pleistocene age) |  | General definitions of E, F and G see D. |
| F | Fan terraces or alluvial fans (alluvium from mixed igneous rock of Holocene or late Pleistocene age) |  |  |
| G | Fan terraces or alluvial fans (alluvium from mixed igneous rock of middle to early Pleistocene age) |  |  |

^*^K = 10^3^ and M = 10^6^ years before present.

**Table S2.** Statistics of time-series woody cover field measures.

| Year | Mean (%) | Standard error | n | Mean elevation (m) |
| --- | --- | --- | --- | --- |
| 1957 | 17.4 | 2.1 | 37 | 1063 |
| 1958 | 19.7 | 2.4 | 37 | 1063 |
| 1959 | 12.9 | 1.5 | 62 | 1111 |
| 1960 | 12.7 | 1.5 | 52 | 1088 |
| 1961 | 12.3 | 1.2 | 62 | 1111 |
| 1962 | 14.3 | 1.4 | 61 | 1111 |
| 1963 | 15.5 | 1.4 | 62 | 1111 |
| 1964 | 18.7 | 1.5 | 62 | 1111 |
| 1965 | 16.9 | 1.5 | 62 | 1111 |
| 1966 | 23.4 | 1.7 | 62 | 1111 |
| 1967 | 20.0 | 2.2 | 37 | 1063 |
| 1968 | 16.3 | 1.5 | 81 | 1130 |
| 1969 | 22.4 | 1.4 | 79 | 1128 |
| 1981 | 26.0 | 1.7 | 80 | 1129 |
| 1984 | 33.9 | 1.9 | 81 | 1130 |
| 1991 | 36.9 | 2.2 | 81 | 1130 |
| 1994 | 27.7 | 1.8 | 78 | 1129 |
| 1997 | 22.8 | 1.5 | 81 | 1130 |
| 2000 | 24.7 | 1.5 | 81 | 1130 |
| 2003 | 21.8 | 1.4 | 81 | 1130 |
| 2006 | 23.5 | 1.4 | 81 | 1130 |
| 2009 | 24.2 | 1.4 | 81 | 1130 |
| 2012 | 23.7 | 1.4 | 81 | 1130 |
